# Measuring personal recovery in people with bipolar disorder and exploring its relationship with well‐being and social role participation

**DOI:** 10.1002/cpp.2371

**Published:** 2019-06-10

**Authors:** Jannis T. Kraiss, Peter M. ten Klooster, Melissa Chrispijn, Anja W.M.M. Stevens, Ralph W. Kupka, Ernst T. Bohlmeijer

**Affiliations:** ^1^ Centre for eHealth and Well‐being Research, Department of Psychology, Health, and Technology University of Twente Enschede Netherlands; ^2^ Center for Bipolar Disorders Dimence Mental Health Deventer Netherlands; ^3^ Amsterdam UMC, Vrije Universiteit, Psychiatry Amsterdam Public Health research institute Amsterdam Netherlands

**Keywords:** bipolar disorder, personal recovery, psychometric evaluation, social role participation, well‐being

## Abstract

The relevance of personal recovery receives increasing attention in mental health care and is also important for people with bipolar disorder (BD). There is a need for reliable and valid instruments measuring personal recovery. Therefore, the current study evaluated the psychometric properties of a Dutch translation of the Questionnaire about the Process of Recovery (QPR) in a sample of people with BD and explored the relationship with constructs of well‐being, social role participation, and psychopathology. A cross‐sectional survey study was conducted in which 102 people diagnosed with BD completed the QPR. Factor structure of the QPR was evaluated by conducting confirmatory factor analyses (CFA), and internal consistency was assessed by calculating reliability coefficients. Convergent validation measures assessed well‐being, social role participation, and symptomatology. Incremental validity was determined by evaluating the ability of the QPR to explain variance in symptomatology above and beyond well‐being. Findings of the CFA supported a unidimensional factor structure, and internal consistency estimates were excellent. Scores of the QPR showed strong correlations with convergent measures, but were only weakly associated with manic symptomatology. Moreover, personal recovery explained additional variance in symptoms of depression and anxiety above and beyond well‐being, indicating incremental validity. The QPR appears to be a reliable and valid tool to assess personal recovery in people with BD. Our findings underline the importance of personal recovery in the context of treatment of BD. Personal recovery demonstrates a substantial overlap with well‐being.

**Key practitioner points**

- The QPR is a reliable and valid tool to assess personal recovery in patients with BD.
- Personal recovery is strongly related with well‐being, social role participation, and symptomatology and may thus be considered as an important outcome in people with BD.
- The assessment of personal recovery has additional value over assessing well‐being and may be useful in the treatment of people with psychiatric disorders.

## 1 INTRODUCTION

Bipolar disorder (BD) is a severe and chronic affective disorder, which characterized by shifting depressive and (hypo)manic mood episodes (Kupka, Knoppert, & Nolen, 2008). In general, a distinction is made between bipolar I (BDI) and bipolar II disorder (BDII). In BDII, an individual has never experienced a full manic episode but only milder hypomanic episode(s) (Grande, Berk, Birmaher, & Vieta, 2015). Prevalence estimates from a large community sample from 11 countries revealed a lifetime prevalence of 0.6% for BDI, 0.4% for BDII, and 1.4% for subthreshold BD (Merikangas et al., 2007). The economic burden of BD was estimated at 151 billion dollars per year in the United States alone (Dilsaver, 2011). Suffering from BD is associated with negative social consequences (Calabrese et al., 2003), decreased quality of life (Dean, Gerner, & Gerner, 2004), work‐related issues (Fajutrao, Locklear, Priaulx, & Heyes, 2009; Laxman, Lovibond, & Hassan, 2008), and a high burden for caregivers (Miller, Dell'Osso, & Ketter, 2014).

In mental health care, the concept of personal recovery is receiving increasing interest (Fava, Ruini, & Belaise, 2007; Jones, Mulligan, Higginson, Dunn, & Morrison, 2013; Leamy, Bird, Le Boutillier, Williams, & Slade, 2011), and especially Anglophone countries move towards supporting personal recovery in the treatment of people with mental disorders (Bird et al., 2014). In contrast to symptomatic and functional recovery, personal recovery has been defined as a “deeply personal, unique process of changing one's attitudes, values, feelings, goals, skills and/or roles [and] a way of living a satisfying, hopeful and contributing life even with the limitations caused by illness” (Leamy et al., 2011, p. 445). Leamy et al. (2011) identified five key processes important for personal recovery: connectedness, hope, identity, meaning, and empowerment. These five processes are comprised in the CHIME framework of personal recovery (Leamy et al., 2011). People with severe mental disorders, such as BD, have highlighted the relevance of hope, meaning, and connectedness in life as important outcomes of recovery in contrast to the traditional focus on symptomatic recovery (Jones et al., 2012; Mead & Copeland, 2000; Pitt, Kilbride, Nothard, Welford, & Morrison, 2007; Slade, 2009).

Independent from but related to the idea of personal recovery, the paradigm of focusing on mental health as opposed to mental illness has also received increased attention. Fava et al. (2007) define full recovery as the absence of psychopathology and the presence of psychological well‐being (Ryff, 2014). In a similar way, Keyes (2005) defines mental health not only merely as the absence of psychopathology but also as the presence of well‐being. In his conceptualization, well‐being comprises three dimensions: (a) emotional (e.g., presence of positive emotions), (b) psychological (e.g., autonomy and environmental mastery), and (c) social well‐being (e.g., social acceptance or social coherence). Research shows that the presence of well‐being buffers against psychopathology (e.g., Keyes, Dhingra, & Simoes, 2010; Lamers, Westerhof, Glas, & Bohlmeijer, 2015; Schotanus‐Dijkstra et al., 2016). Although personal recovery and well‐being emerged as independent concepts, they share substantial conceptual overlap (Slade, 2010). Besides personal recovery and well‐being, social role participation is increasingly considered a key outcome in rehabilitation of people with a wide range of chronic impairments, including those with psychiatric conditions and may be another important factor for recovery (Jaeger & Hoff, 2012; Whitley & Drake, 2010). It has been shown to be important to build and maintain self‐esteem and autonomy (Gordeev et al., 2010) and plays a role in long‐term mental health (Oude Voshaar et al., 2016).

Several measures exist to assess personal recovery. Shanks et al. (2013) systematically reviewed the recovery literature for measures of personal recovery and found 13 questionnaires assessing personal recovery. Of the identified measures, only from the Questionnaire about the Process of Recovery (QPR) all items matched with the processes of the CHIME framework and at the same time covered all five processes. On the basis of service users' accounts of recovery from psychosis, the original QPR comprised 22 items and contained the two dimensions: (a) intrapersonal and (b) interpersonal recovery (Neil et al., 2009). Although originally developed and validated in people with experience of psychosis, all items of the QPR refer to generic, nonpsychosis‐specific processes of recovery that have been identified in the CHIME framework as relevant across mental illnesses.

The original English QPR has been translated and validated in samples with different or mixed mental disorders in several languages, including Chinese (Chien & Chan, 2013), Swedish (Argentzell, Hultqvist, Neil, & Eklund, 2017), and Japanese (Kanehara et al., 2017). Further psychometric analyses by Law, Neil, Dunn, and Morrison (2014) showed that the most interpretable solution of the QPR was a unidimensional 15‐item version. Williams et al. (2015) could confirm this conclusion by comparing the 22‐item version with the 15‐item version. They found that the interpersonal recovery subscale of the 22‐item version underperformed in confirmatory factor analyses (CFA) and that the intrapersonal subscale showed substantial overlap with the 15‐item version. The authors concluded that the 15‐item version was more robust and less burdensome compared with the 22‐item version (Williams et al., 2015). Another specific measure of recovery experiences in BD is the Bipolar Recovery Questionnaire (BRQ; Jones et al., 2013). The BRQ has been developed in a sample of patients with BD and has been shown to be a reliable and valid instrument (Jones et al., 2013). However, the QPR represents a less burdensome and more feasible solution to assess recovery since the BRQ comprises 36 items. Moreover, the BRQ fits less well to the well‐established and evidence‐based CHIME framework compared with the QPR of which every item maps to one of its dimensions (Shanks et al., 2013).

Although the QPR has been validated in several different languages and target groups, there are some important gaps in current knowledge. First, the QPR has not yet been validated in people with BD. The illness course of BD is often chronic (Fagiolini et al., 2013), and although people with BD might recover symptomatically and functionally, this does not necessarily mean that they are personally recovered. The QPR can be a potentially suitable instrument to assess personal recovery in BD since it was the only questionnaire identified of which every item maps a dimension of the CHIME framework (Shanks et al., 2013). Moreover, it has been developed in cooperation with mental health service user's experiences of recovery from psychosis (Neil et al., 2009). Even though it has not been specifically developed with people with BD, many important personal recovery challenges such as meaning or identity can be seen as transdiagnostic across various serious mental illnesses (Jones, Higginson, Murray, & Morrison, 2010; Mead & Copeland, 2000; Pitt et al., 2007). Hence, the QPR may also be appropriate to assess recovery in BD. Second, the relationship between the concepts of personal recovery, well‐being, social role participation, and symptomatology has not yet been explored in people with BD. Third, the QPR has not yet been translated into Dutch. Translating the QPR into Dutch would be an important step for assessment of personal recovery in the Netherlands.

Therefore, the goal of the current study is three fold: (a) to confirm the unidimensional factor structure and internal consistency of a Dutch translation of the QPR, (b) to investigate convergent validity of the QPR with measures of well‐being, social role participation, and psychopathology, and (c) to determine the incremental validity of the QPR in explaining variance in symptoms of anxiety and depression above and beyond scores of well‐being in a sample of people with BD.

For convergent validity, we hypothesized strong positive correlations between personal recovery and well‐being since these two constructs show substantial conceptual convergence (Slade, 2010) and earlier studies have shown strong positive relationships between personal recovery and well‐being related outcomes, such as optimism (Neil et al., 2009) and self‐esteem (Law et al., 2014; Neil et al., 2009) and a moderate positive correlation with well‐being (Williams et al., 2015). In particular, strong positive relationships between personal recovery and the emotional and psychological dimensions of well‐being were expected. For example, similar to emotional and psychological well‐being, personal recovery focuses on an individual's experience of positive emotion and sense of autonomy, self‐acceptance, and meaning. Furthermore, we hypothesized moderate to strong positive correlations between personal recovery and social role participation since social role participation plays an important role in long‐term mental health (Gordeev et al., 2010; Oude Voshaar et al., 2016) and has been widely recognized as important part of recovery (Jaeger & Hoff, 2012; Whitley & Drake, 2010). Finally, we hypothesized moderate to strong negative correlations between personal recovery and symptomatology. This would be in line with an earlier study, showing moderate correlations with hopelessness and strong correlations with depressive symptomatology (Law et al., 2014).

## 2 METHOD

### 2.1 Procedure

A cross‐sectional validation survey study was conducted. Participants were gathered through convenience sampling via the Dutch patient association for BD. The study was promoted in the newsletter and in the journal of the patient association. Data were gathered via the online‐survey tool Limesurvey (https://www.limesurvey.org/). At the beginning of the survey, respondents were informed about the aim of the study, that they could stop the survey at any moment and that their data were processed anonymously and confidentially. Ten shopping vouchers of 50 euro were raffled among all participants. The study was approved by the Ethics Committee of the University of Twente.

### 2.2 Measures

Participants were asked to provide basic demographical data, including gender, age, marital and employment status, ethnicity, and educational background. People were also asked to specify their type of diagnosis (BDI or BDII) and if they were in psychological or psychiatric treatment at the moment of participation. Moreover, they were asked if they were taking medication in the context of their BD and whether there were any recent adaptations in their medication. The following questionnaires were used to assess the relevant constructs.

#### 2.2.1 Personal recovery

For the current study, the 15‐item version of the QPR (Law et al., 2014; Neil et al., 2009) was used to measure personal recovery. The 15 items of the QPR (e.g., “I feel better about myself” or “I can actively engage with life”) can be answered on a 5‐point Likert scale, ranging from 0 (*disagree strongly*) to 4 (*agree strongly*). More personal recovery is indicated by higher scores on the questionnaire. For this study, the QPR was translated from English into Dutch through forward and backward translation by the first and second author. The English 15‐item version of the QPR showed high internal consistency in a sample of psychotic individuals (*α* = .89; Williams et al., 2015) and in a sample of people with schizophrenia spectrum disorder (*α* = .93; Law et al., 2014).

#### 2.2.2 Well‐being

The 14‐item Mental Health Continuum – Short Form (MHC‐SF; Lamers, Westerhof, Bohlmeijer, ten Klooster, & Keyes, 2011) aims to assess well‐being on three dimensions: (a) emotional well‐being (three items), (b) psychological well‐being (six items), and (c) social well‐being (five items). Respondents rate the frequency of feelings on a 6‐point Likert scale in the past month, ranging from 0 (*never*) to 5 (*every day*). We used the Dutch version of the MHC‐SF, which showed high internal consistency for the total scale (*α* = .89) and for the scales emotional (*α* = .83) and psychological well‐being (*α* = .83) and adequate reliability for the social dimension (*α* = .74). The MHC‐SF has also shown convergent validity by correlating well with aspects of well‐being and positive functioning (Lamers et al., 2011). Cronbach's *α* in the current study was .91 for the total scale and .89 and .87 and .67 for the emotional, psychological, and social well‐being subscale, respectively.

#### 2.2.3 Social role participation

The Short Social Role Participation Questionnaire (S‐SRPQ; Oude Voshaar et al., 2016) was used to assess social role participation. The 12‐item S‐SRPQ contains two subscales: (a) satisfaction with role performance and (b) experienced difficulties. Respondents are asked to specify their satisfaction with their social role and experienced difficulties in relation to six social roles (e.g., intimate relationship or employment). The items are scored on a 5‐point Likert scale, reaching from 0 (*not satisfied at all* or *no difficulties at all*) to 4 (*very much satisfied* or *not possible*). More satisfaction and more experienced difficulties are indicated by higher scores on the corresponding subscales. Both subscales of the questionnaire have shown good internal consistency (*α* = .86; Oude Voshaar et al., 2016). Cronbach's *α* in the current study was adequate for the satisfaction subscale (*α* = .74) and good for the experienced difficulties subscale (*α* = .82).

#### 2.2.4 Symptoms of depression and anxiety

To assess depressive and anxious symptomatology, the Hospital Anxiety and Depression Scale (HADS; Zigmond & Snaith, 1983) was used. This 14‐item questionnaire assesses the presence of symptoms of depression (seven items) and anxiety (seven items). Respondents are asked to rate the frequency of symptoms over the last week, with scores ranging from 0 (*not at all*) to 3 (*very often*). Prior psychometric validations have shown good and adequate reliability of the anxiety (*α* = .84) and depression subscale (*α* = .79) of the Dutch HADS in the general population (Spinhoven et al., 1997). In the current study, Cronbach's *α* was .85 and .73 for the anxiety and depression subscale, respectively.

#### 2.2.5 Manic symptomatology

Symptoms of mania were assessed using the Altman Self‐Rating Mania Scale (ASRM; Altman, Hedeker, Peterson, & Davis, 1997). This measure comprises five items assessing manic symptoms (e.g., inflated self‐confidence or increased cheerfulness) over the past week. Each item contains five answering options representing the severity of the symptoms. Higher scores are indicative of more manic symptoms. Prior psychometric evaluations have shown good test–retest reliability (Altman et al., 1997) and sensitivity to change in a clinical group (Altman, Hedeker, Peterson, & Davis, 2001). Cronbach's *α* in the current study was .73.

### 2.3 Statistical analyses

Statistical analyses were performed using Mplus version 7.11 (Muthén & Muthén, 2010), RStudio (R Core Team, 2018), and the statistical package for social sciences version 25 (SPPS). CFA was conducted in Mplus to confirm the previously established unidimensional factor structure of the QPR (Argentzell et al., 2017; Law et al., 2014; Williams et al., 2015). For this, we fitted a strict one‐factor solution in which all 15 items loaded on one single latent factor. We used the robust diagonally weighted least square mean and variance adjusted estimation method, given the relatively small sample size and ordinal nature of the data (Flora & Curran, 2004; Moshagen & Musch, 2014). Factor loadings >0.40 were considered satisfactory (Floyd & Widaman, 1995; Hair, Black, Babin, Anderson, & Tatham, 2009). Chi‐square statistics (*χ* ^*2*^ *)* were used to assess the model fit, where a smaller and nonsignificant *χ* ^*2*^ value is indicative for a better model fit and a ratio between the *χ* ^*2*^ value, and the degrees of freedom should be <5 for an acceptable and around <2 for a good model fit (Kline, 2015; Watkins, 1989). Additionally, we used the comparative fit index (CFI), Tucker‐Lewis Index (TLI), weighted root‐mean‐square residual (WRMR), and root‐mean‐square error approximation (RMSEA) to evaluate the model fit (Hu & Bentler, 1998). Values ≥0.90 are seen as acceptable and ≥ 0.95 as good model fit for the CFI and TLI (Hu & Bentler, 1999). In addition, WRMR values <1 were seen as good model fit (DiStefano, Liu, Jiang, & Shi, 2018; Yu, 2002), and RMSEA values ≤0.08 were considered as acceptable and ≤0.05 as good model fit (Browne & Cudeck, 1992). The evaluation of the model was first based on a restrictive model assuming uncorrelated error terms. Although model fit can often be increased by allowing errors terms to correlate, we decided to only allow error correlation(s) to improve model fit if an initial evaluation of the model indicated unacceptable fit and if it made substantial sense (Jöreskog, 1993). The difference in fit between the 1‐factor model without error correlations and the model where error correlations were allowed, was statically tested with the DIFFTEST function of Mplus, which adequately deals with *χ* ^*2*^ difference testing of nested models.

Internal consistency was determined by calculating Cronbach's *α* and categorical McDonald's omega (ω; Dunn, Baguley, & Brunsden, 2014). Since Cronbach's *α* assumes tau‐equivalence and may thus be deficient for evaluating the internal consistency of congeneric models, McDonalds ω was calculated as an alternative estimate of internal consistency. We used the MBESS package (Kelley, 2018) in RStudio to calculate *α* and categorical ω coefficients with 95% bias corrected and accelerated confidence intervals based on 1,000 bootstrap samples. Estimated values >0.70 were seen as acceptable and >0.80 as good reliability (Cicchetti, 1994). In addition, we calculated item‐total correlations for each item of the QPR.

Construct validity of the QPR was determined by calculating Pearson's correlation coefficients between scores of the QPR and convergent measures of well‐being (MHC‐SF), social role participation (S‐SRPQ), and depressive (HADS‐D), anxious (HADS‐A), and manic symptomatology (ASRM). Values between 0.1 and 0.3 were considered as weak, between 0.3 and 0.5 as moderate correlation, and larger than 0.5 as strong correlation. To determine incremental validity, we conducted two separate multiple hierarchical regression analyses to test the ability of the QPR to explain variance in psychopathology above and beyond well‐being. In the first step, scores of the MHC‐SF were entered, respectively, and in the second step, total scores of the QPR were in entered to the model. Significant F‐changes (*p* < .05) in the second step of the model were indicative for incremental validity of the QPR.

## 3 RESULTS

### 3.1 Description of the sample

Mean age of the 102 respondents was 52 years (*SD* = 11.17, range 23–77). More than three quarters of the sample was female, and half of the sample was married, whereas the rest has never been married or was divorced. A large number of participants were not currently working, and approximately half of the sample had a high educational background. The mean score of the QPR in the current sample was 37.66 (*SD* = 11.14). This is relatively low compared with a prior study by Law et al. (2014) in which they included a mixed sample of mental health service users and found a mean QPR score of 50.13 (*SD* = 11.56). A detailed overview of the sample characteristics can be found in Table 1.

**Table 1:** Sample characteristics (*N* = 102)

		N	%
Gender	Female	80	78.4
	Male	22	21.6
Marital status	Married	53	52.0
	Never married	26	25.5
	Divorced	22	21.6
	Widowed	1	1.0
Employment status	Not capable to work	38	39.6
	Paid work	24	25.0
	Voluntary work	14	14.6
	Retired	9	9.4
	Housewife/houseman	4	4.2
	Self‐employed	4	4.2
	Student	3	3.1
	Other	6	5.9
Education	Low	13	12.9
	Moderate	32	31.7
	High	56	55.4
Diagnosis	BDI	41	40.2
	BDII	47	46.1
	Unknown	14	13.7
Currently in psychological treatment	Yes	84	82.4
	No	18	17.6
Currently taking medication	Yes	97	95.1
	No	5	4.9

### 3.2 Factor structure and internal consistency

The one‐factor solution revealed acceptable CFI (0.964), TLI (0.958), and WRMR values (0.895), and all items showed satisfactory factor loadings. However, RMSEA (0.105) indicated a poor fit to the data. The chi‐square test of model fit was significant (χ2 = 191.11, *df* = 90, *p* < .001), but the ratio between the χ2 value and degrees of freedom was smaller than 3, indicating an acceptable fit. On the basis of the modification indices, we allowed an error correlation between items 3 and 4, which led to a slight improvement of fit indices (CFI = 0.969; TLI = 0.963; RMSEA = 0.098; χ2 = 176.03, *p* < .001). The model in which the error correlation between item 3 and 4 (*r* = .661) was allowed, showed a significantly better fit based on a chi‐square difference test (Δχ^2^ = 15.08, Δdf = 1, *p* < .01). However, since the improvement of fit on the other fit indices was negligible and CFI and TLI fit indices already indicated a good fit without error correlations allowed, we decided to adhere to the restrictive 1‐factor model without error correlations allowed. Internal consistency of the QPR was excellent in the present sample (*α* = .92 and ω = 0.95), and corrected item‐total correlations were high for all items. Table 2 gives an overview of the standardized factor loadings, reliability parameters, and corrected item‐total correlations.

**Table 2:** Standardized factor loadings and corrected item‐total correlations for the 15 items of the Questionnaire about the Process of Recovery (QPR)

Item	Factor loading	Corrected item‐total correlation
QPR1 I feel better about myself.	0.79	0.70
QPR2 I feel able to take chances in life.	0.86	0.75
QPR3 I am able to develop positive relationships with other people.	0.83	0.71
QPR4 I feel part of society rather than isolated.	0.80	0.69
QPR5 I am able to assert myself.	0.64	0.55
QPR6 I feel that my life has a purpose.	0.80	0.75
QPR7 My experiences have changed me for the better.	0.70	0.65
QPR8 I have been able to come to terms with things that have happened to me in the past and move on with my life.	0.71	0.66
QPR9 I am basically strongly motivated to get better.	0.49	0.42
QPR10 I can recognize the positive things I have done.	0.65	0.54
QPR11 I am able to understand myself better.	0.65	0.55
QPR12 I can take charge of my life.	0.84	0.78
QPR13 I can actively engage with life.	0.90	0.81
QPR14 I can take control of aspects of my life.	0.85	0.73
QPR15 I can find the time to do the things I enjoy.	0.62	0.56
McDonald's omega (95% CI)	0.95 (0.91–0.97)	
Cronbach's alpha (95% CI)	0.92 (0.90–0.93)	

Abbreviation: CI, confidence interval.

*N* = 102.

### 3.3 Convergent validity

A detailed overview of the descriptive statistics of the validation measures and correlations with the QPR can be found in Table 3. We found a particularly strong relationship between personal recovery and emotional (*r* = .77) and psychological well‐being (*r* = .80) and also with total scores of the MHC‐SF. In addition, we found a lower but still strong positive relationship with social well‐being (*r* = .58). Moreover, a strong positive relationship was found with satisfaction with role performance of the S‐SRPQ (*r* = .63) and a strong negative relationship with experienced difficulty with a social role (*r* = −.53). QPR scores were strongly negatively correlated with depressive symptoms (*r* = −.71) and with anxious symptomatology (*r* = −.50). Finally, a weak negative but significant relationship between the QPR and manic symptoms was found (*r* = −.21).

**Table 3:** Descriptive statistics and bivariate Pearson's correlations between the Questionnaire about the Process of Recovery (QPR) and criterion measures

Measure	M (*SD*)	QPR
QPR (*n* = 102)	37.66 (11.14)	‐
MHC‐SF (*n* = 102)		
Emotional well‐being	7.40 (3.96)	0.77**
Social well‐being	8.36 (4.94)	0.58**
Psychological well‐being	13.58 (7.19)	0.80**
Total score	29.34 (14.48)	0.80**
S‐SRPQ (*n* = 98)		
Satisfaction with role	14.62 (5.41)	0.63**
Experienced difficulty	16.77 (5.41)	−0.53**
HADS (*n* = 98)		
Anxiety symptoms	8.71 (4.64)	−0.50**
Depression symptoms	9.61 (3.99)	−0.71**
ASRM (*n* = 98)		
Total score	2.99 (3.23)	−0.21*

Variations in *n* due to missing data.

Abbreviations: ASRM, Altman Self‐Rating Mania Scale; HADS, Hospital Anxiety and Depression Scale; MHC‐SF, Mental Health Continuum – Short Form; S‐SRPQ, Short version of the Social Role Participation Questionnaire.

* **

### 3.4 Incremental validity

To investigate the incremental validity of the QPR, we tested whether scores of the QPR explained a significant amount variability in psychopathology above and beyond scores of well‐being. Therefore, two separate multiple hierarchical regression analyses with scores of psychopathology as criterion variables were conducted. In the first step, subscale scores of the MHC‐SF were entered. In the second step, scores of the QPR were entered.

In the first model, scores of the QPR explained 3% additional variance in depressive symptoms above and beyond well‐being (*p* < .01), and personal recovery significantly explained depressive symptoms above and beyond well‐being. In the second model, scores of the QPR explained 4% additional variance in anxiety symptoms (*p* < .05) above and beyond well‐being. After controlling for well‐being, personal recovery was uniquely related to anxiety symptoms (*p* < .05). An overview of the regression analyses can be found in Table 4 and Table 5.

**Table 4:** Summary of hierarchical regression analysis for Mental Health Continuum – Short Form (MHC‐SF) subscales and Questionnaire about the Process of Recovery (QPR) and depressive symptoms Hospital Anxiety and Depression Scale – depression subscale (HADS‐D)

Variable	*B*	*SE*	*β*	*t*	*ΔR* ^2^
Step 1					
Constant	15.53	0.61		25.66***	.59***
Emotional well‐being (MHC‐SF)	−0.66	0.10	−.65	−6.31***	
Psychological well‐being (MHC‐SF)	−0.09	0.07	−.16	−1.23	
Social well‐being (MHC‐SF)	0.02	0.08	.02	0.24	
Step 2					
Constant	17.65	0.99		17.91***	.03**
Emotional well‐being (MHC‐SF)	−0.54	0.11	−.54	−4.89***	
Psychological well‐being (MHC ‐SF)	0.00	0.08	.00	0.01	
Social well‐being (MHC‐SF)	0.02	0.08	.02	0.21	
Personal recovery (QPR)	−0.11	0.04	−.31	−2.68**	

** *p* < .01, ***

**Table 5:** Summary of hierarchical regression analysis for Mental Health Continuum – Short Form (MHC‐SF) subscales and Questionnaire about the Process of Recovery (QPR) and anxiety symptoms Hospital Anxiety and Depression Scale – depression subscale (HADS‐A)

Variable	*B*	*SE*	*β*	*t*	*ΔR* ^2^
Step 1					
Constant	12.52	0.97		12.86***	.21***
Emotional well‐being (MHC‐SF)	−0.39	0.17	−.33	−2.31*	
Psychological well‐being (MHC‐SF)	−0.17	0.11	−.26	−1.50	
Social well‐being (MHC‐SF)	0.16	0.12	.18	1.32	
Step 2					
Constant	15.52	1.60		9.71***	.04*
Emotional well‐being (MHC‐SF)	−0.22	0.18	−.19	−1.23	
Psychological well‐being (MHC ‐SF)	−0.05	0.12	−.07	−0.37	
Social well‐being (MHC‐SF)	0.16	0.12	.17	1.32	
Personal recovery (QPR)	−0.16	0.07	−.38	−2.34*	

* *p* < .05,

*** *p* < .001.

## 4 DISCUSSION

The current study is the first to evaluate the psychometric properties of the QPR in a sample of people with BD and to assess the relationship of personal recovery with well‐being, social role participation, and psychopathology. Several measures exist to assess personal recovery. In a recent review, Shanks et al. (2013) identified only one questionnaire assessing all processes of the CHIME framework of personal recovery, namely, the QPR. Originally designed as a 22‐item questionnaire (Neil et al., 2009), the QPR was recently shortened to a 15‐item version (Law et al., 2014; Williams et al., 2015). The QPR has been translated into several languages, including Swedish (Argentzell et al., 2017), Chinese (Chien & Chan, 2013), and Japanese (Kanehara et al., 2017). However, to date, the QPR had not been translated into Dutch and has not been validated in people with BD.

Overall, our findings provide preliminary support for the reliability and validity of the Dutch QPR for measuring personal recovery in people with BD. Findings of the CFA revealed a good fit for a unidimensional model, based on several fit indices. This is in line with studies investigating the 1‐factor model of the QPR (Law et al., 2014; Williams et al., 2015). Furthermore, all items showed high factor loadings. This supports the conclusion of prior studies suggesting an overall recovery score rather than distinct subscales (Law et al., 2014; Williams et al., 2015). The RMSEA fit statistic did not meet the cut‐off for acceptable model fit. It should be noted though that fit indices may not perform uniformly across different conditions. Different factors, such as sample size or parameter estimation methods can affect fit indices in different ways (Cook, Kallen, & Amtmann, 2009). Since all the other fit indices indicated a good model fit, we concluded that there was sufficient support for a unidimensional factor structure overall. Reliability values, including Cronbach's *α* and McDonald's omega were excellent, coinciding with earlier studies (Law et al., 2014; Williams et al., 2015).

QPR scores were significantly related with each validation measure in this study. Most of these relationships were in line with our hypotheses, such as the strong positive correlation between personal recovery and well‐being. This strong relationship can be explained by the high conceptual overlap between well‐being and personal recovery. Slade (2010) outlined the similarity between these two concepts and how they can complement each other. Furthermore, a strong relationship between personal recovery and the two subscales of social role participation was found. This supports the idea that social role participation (e.g., being able to work) can be seen as a relevant factor for recovery (Jaeger & Hoff, 2012; Whitley & Drake, 2010).

Findings regarding the relationship between personal recovery and symptomatology were mixed. We found a strong negative relationship between personal recovery and both symptoms of anxiety and depressive symptoms, which is in line with earlier studies (Law et al., 2014; Neil et al., 2009). This may imply that either personal recovery will yield further symptom reduction or that symptom reduction yields personal recovery. Surprisingly, the relationship between personal recovery and symptoms of mania was only weak. One possible explanation might be that the presence of manic symptoms might not necessarily be an obstacle for personal recovery. Possibly, manic symptoms might actually increase the experience of personal recovery. Another explanation might be the positively skewed and relatively low average ASRM scores in the present sample that might have suppressed the correlation with personal recovery.

Results of the multiple hierarchical regression analyses suggest incremental validity of the QPR, which explained variance in symptoms of depression and anxiety above and beyond well‐being. This is a surprising finding because research indicates that well‐being already is a strong predictor of symptomatology (Wood & Joseph, 2010; Wood, Maltby, Gillett, Linley, & Joseph, 2008), and we also found very strong relationships between well‐being and personal recovery in the present study. This finding is in line with the conceptual overlap between personal recovery and well‐being (Slade, 2010). Although the total variance in depressive and anxious symptomatology only marginally increased in the second step of the models (3% and 4%, respectively), the variance explained by well‐being substantially decreased when adding personal recovery to the model, and the QPR remained independently associated with symptoms. In other words, although there is a strong overlap between these concepts, personal recovery appears to be sufficiently distinct to warrant assessment.

### 4.1 Limitations and future research

Our study also has several limitations that should be considered. First, we used a cross‐sectional design and thus cannot make any inferences about the longitudinal relationship of the included constructs, and we could not evaluate psychometric properties such as sensitivity to change and test–retest reliability of the QPR. Future research could focus on the longitudinal relationships between personal recovery, well‐being, and symptomatology and could evaluate psychometric properties such as sensitivity to change and test–retest reliability of the QPR. Especially the relationship between personal recovery and well‐being might be interesting to further clarify the differences and commonalities between these two concepts. Second, we used a relatively small sample especially for factor analysis purposes and multivariate regression analyses. For example, following recommendations by Hu and Bentler (1999) to include at least 10 participants per free parameter in the model, 150 people would be needed for the 1‐factor model evaluated in the present study. Thus, these results should be interpreted with some caution. It must be noted though, that our sample contains a clinical group and provides the first evaluation of the Dutch QPR. However, future research should try to evaluate the psychometric properties of the Dutch QPR in larger clinical samples. Third, diagnosis of BD was based on self‐report only, and we did not confirm the diagnosis based on a structured clinical interview. However, participants were recruited via the patient association for BD and 95% of the sample stated that they were taking medication in the context of their BD. It can thus be assumed that the vast majority of the sample actually had BD.

### 4.2 Practical and scientific implications

Several implications for both clinical practice and research arise from these findings. Our results give a first indication that the Dutch QPR is a reliable and valid tool and appears to be a promising instrument to assess personal recovery in BD. BD is a prevalent and highly disabling condition in which the concept of personal recovery is particularly important because the course of their disorder is often chronic and recurrent (Fagiolini et al., 2013). Moreover, patients with serious mental illness, such as BD, express the need for personal recovery outcomes (de Vos et al., 2017; Jones et al., 2012; Mead & Copeland, 2000; Pitt et al., 2007; Slade, 2009). Therefore, one of the main advantages of using the QPR in clinical practice is to foster collaboration and improve engagement by demonstrating to the patient that personal recovery is part of the recovery process (Neil et al., 2009). Although scores of the QPR should be interpreted as one overall recovery score rather than distinct subscores for assessment or monitoring purposes (Law et al., 2014; Williams et al., 2015), the QPR might also be used to individually tailor the recovery process and find out which aspects of recovery are important for the patient (Neil et al., 2009). This step is important because the recovery process should be seen as highly individual and unique process (Leamy et al., 2011) in which therapists should pay attention to the individual needs of the patient. In this context, the QPR can also be used to set individual treatment goals (Neil et al., 2009). The current study now also provides the opportunity to use the QPR for above named purposes in the Netherlands. In a research context, the QPR provides standardized scores, which gives the opportunity to compare the effectiveness of interventions across different studies. Law et al. (2014) suggest that a medium effect size of 0.4 would be equivalent to a change of 4.63 points on the overall 15‐item QPR score that is comparable with the findings in the present sample. Trials focusing on personal recovery in the future could thus use the QPR as outcome measure and contribute to the body of recovery research.

## 5 CONCLUSION

The present study suggests that the QPR is a reliable and valid tool to assess personal recovery in people with BD. Although personal recovery seems to have much overlap with well‐being, it appears to be uniquely related with measures of symptomatology. The QPR can be used in both a clinical and research context to assess personal recovery and can be used by clinicians as a tool to improve the process of personal recovery.

## CONFLICT OF INTEREST

No authors of this paper have any conflicts of interest.

## Footnotes

*
*p* < .05,

**
*p* < .01.

***
*p* < .001.
